# Age-related changes in gut microbiota composition from newborn to centenarian: a cross-sectional study

**DOI:** 10.1186/s12866-016-0708-5

**Published:** 2016-05-25

**Authors:** Toshitaka Odamaki, Kumiko Kato, Hirosuke Sugahara, Nanami Hashikura, Sachiko Takahashi, Jin-zhong Xiao, Fumiaki Abe, Ro Osawa

**Affiliations:** Next Generation Science Institute, Morinaga Milk Industry Co., Ltd., Zama, Kanagawa Japan; Food Ingredients & Technology Institute, Morinaga Milk Industry Co., Ltd., Zama, Kanagawa Japan; Department of Bioresource Science, Graduate School of Agricultural Science, Kobe University, Kobe, Hyogo Japan

**Keywords:** Gut microbiota, Commensal bacteria, Japanese population, Aging

## Abstract

**Background:**

It has been reported that the composition of human gut microbiota changes with age; however, few studies have used molecular techniques to investigate the long-term, sequential changes in gut microbiota composition. In this study, we investigated the sequential changes in gut microbiota composition in newborn to centenarian Japanese subjects.

**Results:**

Fecal samples from 367 healthy Japanese subjects between the ages of 0 and 104 years were analyzed by high-throughput sequencing of amplicons derived from the V3-V4 region of the 16S rRNA gene. Analysis based on bacterial co-abundance groups (CAGs) defined by Kendall correlations between genera revealed that certain transition types of microbiota were enriched in infants, adults, elderly individuals and both infant and elderly subjects. More positive correlations between the relative abundances of genera were observed in the elderly-associated CAGs compared with the infant- and adult-associated CAGs. Hierarchical Ward’s linkage clustering based on the abundance of genera indicated five clusters, with median (interquartile range) ages of 3 (0–35), 33 (24–45), 42 (32–62), 77 (36–84) and 94 (86–98) years. Subjects were predominantly clustered with their matched age; however, some of them fell into mismatched age clusters. Furthermore, clustering based on the proportion of transporters predicted by phylogenetic investigation of communities by reconstruction of unobserved states (PICRUSt) showed that subjects were divided into two age-related groups, the adult-enriched and infant/elderly-enriched clusters. Notably, all the drug transporters based on Kyoto Encyclopedia of Genes and Genomes (KEGG) Orthology groups were found in the infant/elderly-enriched cluster.

**Conclusion:**

Our results indicate some patterns and transition points in the compositional changes in gut microbiota with age. In addition, the transporter property prediction results suggest that nutrients in the gut might play an important role in changing the gut microbiota composition with age.

**Electronic supplementary material:**

The online version of this article (doi:10.1186/s12866-016-0708-5) contains supplementary material, which is available to authorized users.

## Background

The microbiota composition of the human gut changes with age, and alterations in this composition influence human health. In the early 1970s, culture-based methods were used to demonstrate that the gut microbiota composition changes during the aging process [1]. Recent studies using molecular methods have also indicated clear differences in the composition of gut microbiota among infants, toddlers, adults and the elderly [2]. After birth, the initial microbiota composition is affected by the mode of birth [3–5] and the mother’s gut microbiota [6, 7]. Subsequently, a significant shift in the composition of the gut microbe community occurs when the infant transitions to a more solid and varied diet. A recent report suggested that the age-affiliated microbiota population shifts from 3 days to 2 years after birth and that major differences are apparent between 2 years and adulthood [8]. Other reports have indicated that the phylogenetic composition of the bacterial communities evolve towards an adult-like configuration within the 3- [9] or 4-year [10] period after birth. It has been recently shown that the gut microbiota is not yet established at 5 years of age [11]. Another broad shift in gut microbe populations occurs later in life. However, almost all studies related to the gut microbiota of the elderly have been performed on subjects classified into segmented age groups based on varying definitions of ‘elderly’, such as ‘over 60’ [12], ‘over 65’ [13], ‘over 70’ [14] or ‘centenarian’ [15]. It is unclear when and how the microbiota composition shifts from the adult stage to the elderly stage. Yatsunenko et al. [9] conducted a large study with subjects aged 0–83 years and revealed the sequential changes that occur with age. Their report provided important insights, such as the period required to form an adult-like gut microbiota, greater between-subject variation among children than adults, differences in the phylogenetic composition of gut microbiota among individuals from different countries and an increase in bacterial diversity with age. Nevertheless, the sequential changes that occur in the elderly remained unclear due to the limited number of subjects older than 60 years. Furthermore, although abundant data on gut microbiota composition are available in some public databases, sequential changes cannot be evaluated with these public data because of biases stemming from differences in study methods, especially in DNA extraction [16, 17], and nationality differences among the subjects [9, 18–22].

We identified the sequential changes in gut microbiota composition in Japanese subjects over a wide age range, 0–104 years. Our results provide new insights into the developmental period for gut microbiota composition and the patterns of change with age.

## Results

### Overview of gut microbiota composition in each age group

**Fig. 1:**
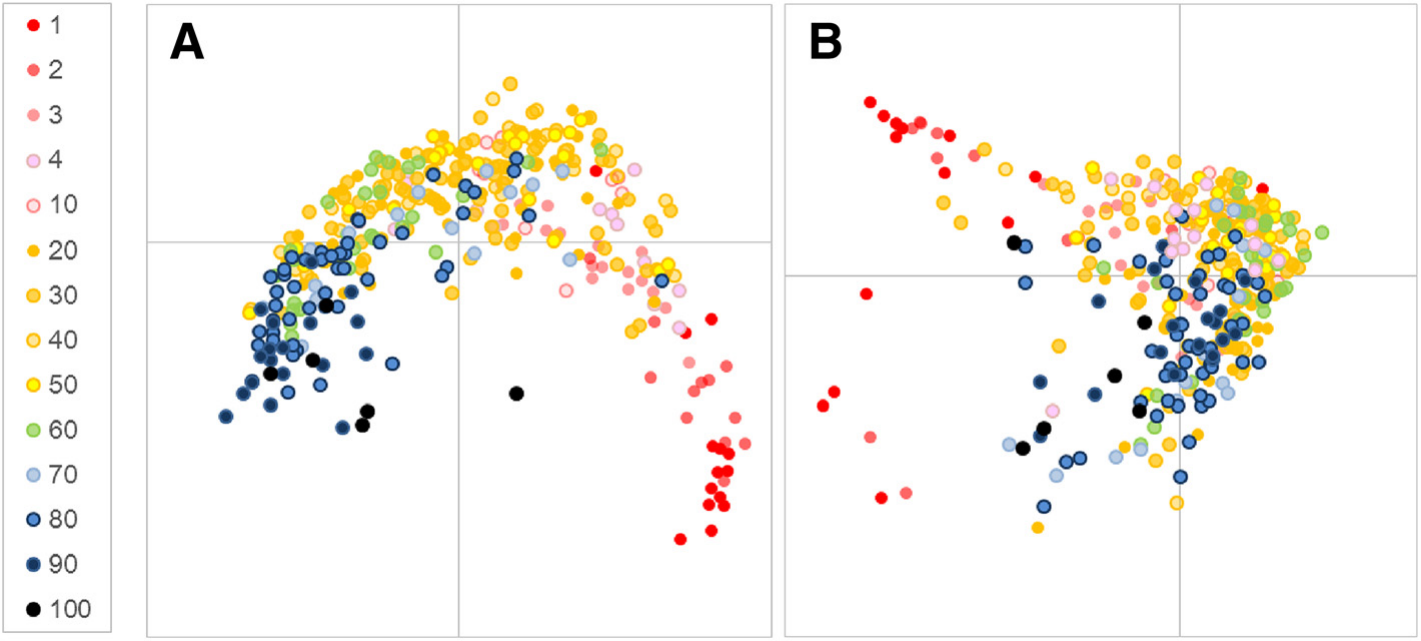
UniFrac clustering for each age group. **a** Unweighted and **b** weighted UniFrac PCoA of gut microbiota from 371 samples collected from the infant to the centenarian stage. Each number in the legend indicates a group as shown in Table 1

A total of 1,839,703 high-quality paired sequences were obtained from the 371 samples, with 4,959 ± 1,813 (average ± standard deviation) reads per sample, which were clustered into 5,952 OTUs and classified into 187 bacterial groups at the genus level (186 genera and one unidentified group). We first calculated UniFrac distances to determine the extent of similarity between microbial communities. UniFrac PCoA (principal co-ordinate analysis) of 5,952 OTUs indicated that age explained the variation in our data set using both weighted and un-weighted analyses (Fig. 1). No gender differences were observed (Additional file 1).

**Fig. 2:**
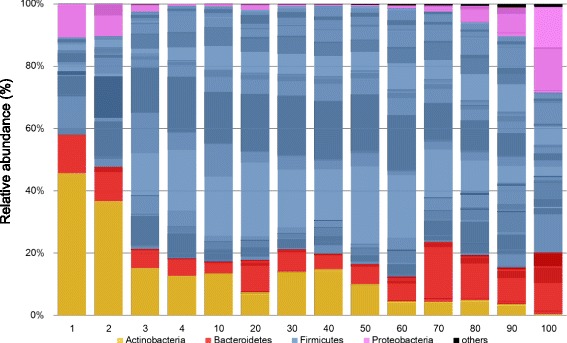
Age-related sequential changes in gut microbiota composition. Overview of phylum/genus composition. Orange, Actinobacteria; Blue, Firmicutes; Red, Bacteroidetes; Pink, Proteobacteria; Black, sum of other phyla. Each component of the cumulative bar chart indicates a genus. Each number indicates a group as shown in Table 1

The phylum and genus compositions of gut microbiota in each age group are shown in Fig. 2, Additional file 2 and Additional file 3. In agreement with previous results, the microbiota composition included four predominant phyla. The relative abundance of Actinobacteria substantially decreased after weaning and continued to decrease with age. Firmicutes was the most predominant phylum after weaning but was less abundant in children younger than 4 years compared with subjects older than 4 years. Increases in the relative abundance of Bacteroidetes and Proteobacteria were observed in subjects over 70 years old. The relative abundance of Bacteroidetes did not change sequentially, but a stepwise increase was observed beyond 70 years of age. The change in the relative abundance of Proteobacteria was opposite that of Firmicutes.

### Age-related changes in genera and their correlations

**Fig. 3:**
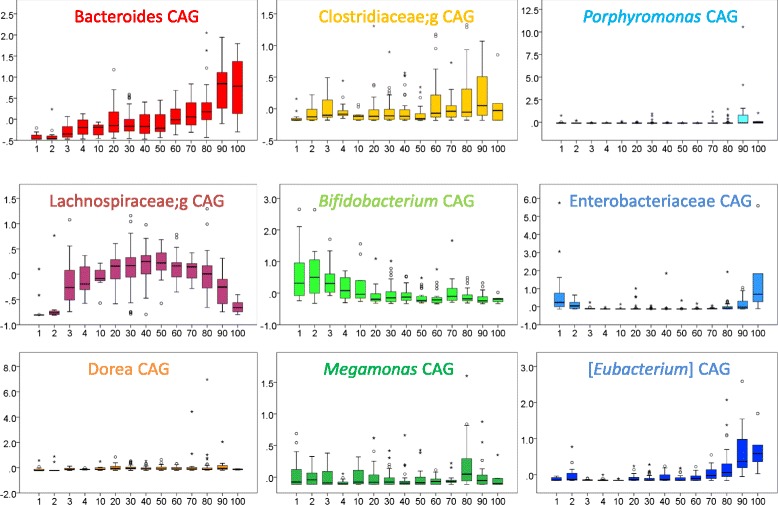
Transition type of each co-abundance group (CAG) from infant to centenarian. Each number indicates a group as shown in Table 1. Box-plots show the interquartile range (IQR) of the sum of z-scores converted from the relative abundance of genera belonging to the same CAG. Open circles and asterisks indicate outliers from 1.5- to 3.0-fold IQR and over 3.0-fold IQR, respectively

**Fig. 4:**
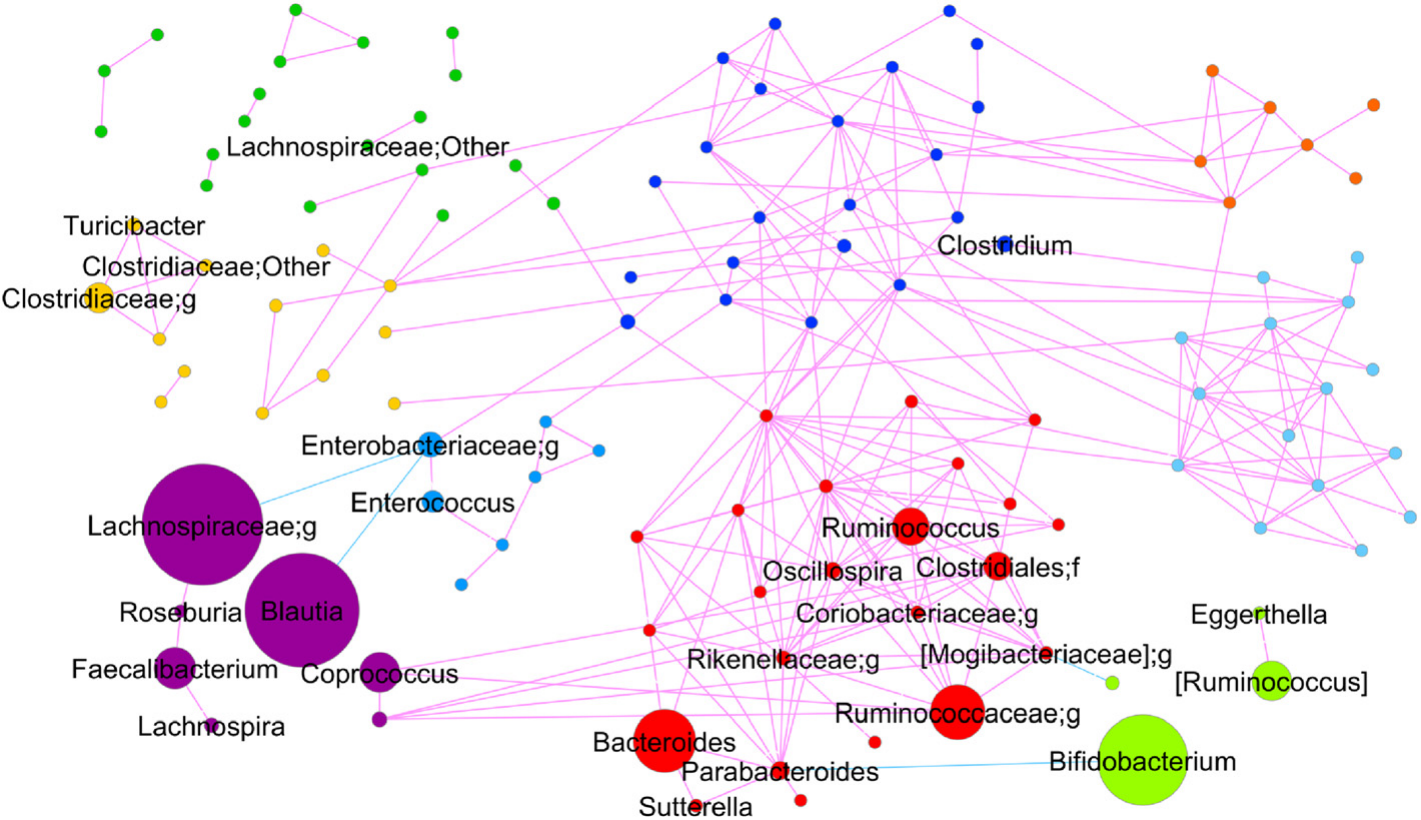
Network plot highlighting relationships between genera in nine CAGs. The colors of each node indicate the nine CAGs as shown in Fig. 3. Circle size indicates genus abundance. Pink and blue lines show significant positive and negative correlations between two bacterial genera with an absolute coefficient value greater than 0.3. Taxa that are found in more than 50 % of the subjects were indicated

To explore the changes in gut microbiota with age in detail, we calculated the co-abundance associations of genera and then clustered the correlated genera into nine CAGs (Additional file 4), which describe the significant differences in microbiota structure among subject groups (Permutational MANOVA, *p* < 0.001). The transition from infant to centenarian was accompanied by distinctive CAG dominance, with a significant abundance of *Bacteroides*, [*Eubacterium*] and Clostridiaceae CAGs (elderly-associated CAGs); Enterobacteriaceae CAGs (infant and elderly-associated CAGs); *Bifidobacterium* CAGs (infant/child-associated CAGs); and Lachnospiraceae CAGs (adult-associated CAGs) (Fig. 3). *Megamonas* and *Peptoniphilus* CAGs were relatively enriched in the elderly. *Dorea* CAG abundance appeared unrelated to aging. Sequential changes occurred in the relative abundance of *Bacteroides,* Lachnospiraceae and *Bifidobacterium* CAGs in the gut microbiota during childhood and adolescence. Not all CAGs were composed exclusively of related species, as shown in Additional files 5 and 6. Wiggum plots showed the relative abundance of each genus and significant associations between nine CAGs (Fig. 4, Additional file 5). Among the 186 genera, 116 had associations with other genera with an absolute coefficient value >0.3 (Additional file 5 and 6 and Fig. 4). Almost all of the correlations were positive; the only four negative correlations were observed between Enterobacteriaceae and Lachnospiraceae, Enterobacteriaceae and *Blautia*, *Bifidobacterium* and *Parabacteroides* and *Veillonella* and [Mogibacteriaceae]. A greater number of positive correlations were observed in the elderly-associated CAGs, especially the *Bacteroides* and [*Eubacterium*] CAGs. We then performed in vitro assays to investigate some relevant relationships among genera. In accordance with the Wiggum plot results, both of *Parabacteroides distasonis* JCM 5825 and *Bifidobacterium longum* JCM 1217 growth were suppressed when co-cultivate with each other (Additional file 7). A similar negative relationship was observed between *Escherichia coli* JCM 1649, belong to family Enterobacteriaceae and *Blautia producta* JCM 1471. In contrast, *Bacteroides uniformis* JCM 5828 growth increased when co-cultivated with *Parabacteroides distasonis* JCM 5825.

**Fig. 5:**
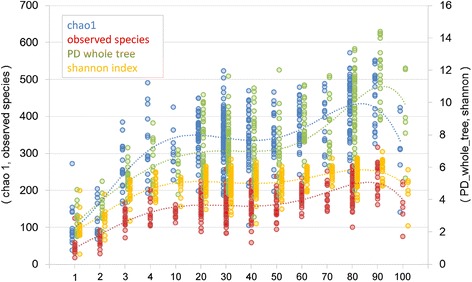
Age-related change in alpha-diversities of gut microbiota. Dashed line indicates a polynomial approximation for each alpha-diversity. Each number below the figure indicates a group as shown in Table 1

As for gut microbiota diversity, all four alpha diversity scores based on PD whole tree, Chao1, the number of observed species and the Shannon index substantially increased after weaning and continued to increase sequentially until the twenties. These scores were stable during adulthood and then increased again at the elderly stage until the centenarian stage (Fig. 5).

### Period of age-related change in community structure of gut microbiota

We performed hierarchical Ward’s linkage clustering based on the abundance of genus-like groups to predict the period of age-related reshaping of gut microbiota. Genus-like groups belonging to the same phylum did not comprise the same cluster, suggesting that the age-related pattern of change was different between the phylum and genus levels. The subjects were divided into three age clusters, for infant, adult and elderly cluster (Additional file 8). The relative abundance of Actinobacteria and Clostridia were significantly higher in infant and adult cluster, respectively (Additional file 9). The elderly cluster showed the significantly higher abundance of Bacteroidetes, Betaproteobacteria and Deltaproteobacteria (Additional file 9). When the subjects were divided into five age clusters, the median (interquartile range) age was 3 (0.5–35) in the infant cluster, 33 (24.75–45.5) in adult cluster I, 42 (32–62) in adult cluster II, 74 (34–81.5) in elderly cluster I and 93.5 (85–98) in elderly cluster II (Additional file 8). The age distributions were similar between the adult I and II clusters and between the elderly I and II clusters. The adult clusters and the elderly clusters showed significant differences in the relative abundance of 32 and 28, respectively, of the 61 genus-like groups, which were found in 50 % of the subjects in any cluster (Additional files 10 and 11). Subjects were predominantly clustered by age; however, some subjects, but not those in the elderly II cluster, fell into a mismatched-age cluster (Additional files 8 and 12).

### Functional properties predicted by PICRUSt

We performed PICRUSt analysis to predict the relative abundance of transporter genes because an altered diet [2] and antibiotic treatment [31] have been reported to be among the most powerful factors that affect the gut microbiota. Clustering based on the relative abundance of the predicted transporters showed that subjects were divided into two age-related groups, the adult-enriched and infant/elderly-enriched clusters (Additional file 13). For example, there was a lower abundance of a predicted xylose transporter (KEGG module: M00215) in pre-weaned infants, probably reflecting the different dietary habits of subjects in each segmented age group (Additional file 14). Interestingly, all drug transporters based on KEGG Orthology groups were found in the infant/elderly-enriched cluster (Additional files 13 and 15).

## Discussion

The composition of gut microbiota is thought to change during the aging process [1]; however, few reports have utilized molecular techniques to investigate the long-term, sequential changes in gut microbiota composition. Our results are in agreement with those of recent studies indicating clear differences in gut microbiota composition among infants, adults and the elderly [2, 23]. The present study using LEfSe method indicated that Actinobacteria, Clostridia and Bacteroidetes, Betaproteobacteria and Deltaproteobacteria were representative taxa in infant, adults and the elderly cluster. Additionally, our results revealed the sequential changes that occur with age from newborns to centenarians. Furthermore, our results showed that Japanese adults (21–69 years old) have a greater abundance of the genera *Blautia* and *Bifidobacterium* (interquartile ranges (IQR) of 18 (12–24) % and 7 (2–14) %, respectively) and a relatively lower abundance of genera related to Bacteroidetes (IQR 4 (1–10) %), compared with those reported in previous studies in other nations. For example, the estimated abundance of *Blautia*, *Bifidobacterium* and Bacteroidetes were < 10 %, < 2 % and > 10 %, respectively, in US and Colombia [19], Korea, China and US [20] and Ireland [30]. These patterns might be characteristic of the gut microbiota composition of the Japanese population, although they may also reflect the DNA extraction method [16, 17] and the amplified region of the 16S rRNA gene [24].

Although there are differences among individuals, our analysis of the phylum composition of gut microbiota in each age group showed a significant shift in the relative abundance of Actinobacteria in infants from before to after weaning. The compositional pattern of gut microbiota during childhood has been thought to impact health later in life [11, 25], but children older than 2 years have not been sufficiently investigated. Our data show that some genera belonging to *Bacteroides,* Lachnospiraceae and *Bifidobacterium* CAGs and the alpha diversity of gut microbiota continued to change sequentially with age in subjects younger than twenty, reflecting the human gut microbiota maturation process. However, children younger than 20 years fell into both the infant and adult clusters, regardless of their age, when clustered based on the abundance of genus-like groups, thus illustrating the individual differences in the gut microbiota maturation process.

In the present study, the Wiggum plot showed negative relationships between the relative abundance of Enterobacteriaceae, which creates an greater endotoxin challenge for the weakened intestinal barrier and thus results in increased stimulation of the inflammatory response [26], and the abundance of *Blautia* and Lachnospiraceae;g (Fig. 4), which belonged to the adult-associated CAG (Lachnospiraceae;g CAG). Other butyrate-producing bacteria, such as *Coprococcus*, *Roseburia* and *Faecalibacterium*, were also clustered in the same CAG. Furusawa et al. reported that microbial-derived butyrate regulates Treg cell differentiation in vitro and in vivo [27]. Given the age-related reduction in the abundance of the genus *Bifidobacterium*, which down-regulates pro-inflammatory responses in the gut epithelium [28–30], our results suggested that the aging-related dysbiosis in elderly subjects may be a contributing factor to inflammatory responses that occur with advancing age.

We performed in vitro assays to investigate bacterial interactions, including those between Enterobacteriaceae and *Blautia*. These results were in accordance with the Wiggum plot results. However, these results might have some biases from the different of environmental condition between in human gut and in vitro assay. In addition, it is uncertain that all bacterial interactions are consistent with the relationships in the Wiggum plot, because genera with positive relationships in the Wiggum plot might grow well under the same environmental conditions without a mutualistic relationship. In contrast, some genera combinations have been reported to exhibit mutualistic relationships,although the absolute values of the correlation coefficients were below 0.3 (no visible relationship in the Wiggum plot). Pande et al. revealed that *Acinetobacter baylyi* and *Escherichia coli* reciprocally exchange essential amino acids [31]. It has also been reported that *Bifidobacterium* populations can be stimulated efficiently with a concomitant decrease in Enterobacteriaceae [32, 33]. Acetate, one of the main fermentation products of *Bifidobacterium,* was reported to promote the growth of butyrate-producing bacteria and the in vitro production of butyrate [34, 35]. Furthermore, *Bifidobacterium longum* has been reported to alter gut luminal metabolism via interactions with *Bacteroides caccae* and *Eubacterium rectale* [36]. Considering these reports, the correlations between gut microbiota members might be more complicated than shown in the Wiggum plot. Our computational analysis results must be interpreted cautiously because they are based on a limited data set. An advanced culture method is needed to clarify the relationships among gut microbiota.

A wide diversity of microorganisms is needed to utilize the many nutrients in adult diets [37]. In addition, a low gut microbiota diversity has been associated with an increasing number of conditions, such as autism [38], autoimmune disease [39] and obesity [40]. Maintaining sufficient bacterial richness and diversity is important for providing gut microbiota with functional redundancy, adaptability and thus systematic robustness against environmental challenges [41]. In this study, we observed an increase in gut microbiota diversity with aging until the centenarian stage. Claesson et al. reported that the alpha diversity of the gut microbiota in community dwellers was significantly higher than that of people in long-stay care [42]. Therefore, in the present study, the observed increase in microbiota diversity with age was likely due to the inclusion of community-dwelling elderly participants. In contrast, centenarians in a Chinese longevous village population had a more diverse gut microbiota than did younger elderly aged 85–99 years [43]. It is uncertain why the trend in diversity differed between Chinese and Japanese centenarians.

Biagi et al. [44] showed a significantly compromised gut microbiota in centenarians but not in elderly subjects aged approximately 70 years compared with a group of younger adults. The authors therefore suggested that a healthy gut microbiota community might be affected by aging-related physiological and behavioral changes that occur after 70 years of age, which was considered the threshold age for defining an individual as elderly. In agreement with this hypothesis, our clustering data suggested that most subjects over 70 years of age comprised two elderly-type clusters (Additional file 8). Nevertheless, the distribution of subjects was not clear from the classification based on subject age. This result suggested that other factors besides age contribute to the composition of gut microbiota communities.

Our study did not allow us to determine why gut microbiota changed with age because we lacked lifestyle and dietary habit information on the subjects. To begin to address this question, we performed PICRUSt analysis to predict the relative abundance of transporter genes because we hypothesized that bacterial transporters that incorporate nutrients from the gut environment would differ if dietary habit was a predominant contributor to gut microbiota composition. Clustering based on the relative abundance of the predicted transporters showed that subjects were divided into two age-related groups, the adult-enriched and infant/elderly-enriched clusters, implying that nutrients in the gut evoke the different gut microbiota compositions between adult and infant/elderly subjects. Not all transporters can explain the relationship with the dietary habits of subjects in each segmented age group, but, for example, the increase in the relative abundance of predicted xylose transporters in subjects after weaning seems to reflect the change from mother’s milk to an omnivorous diet. Interestingly, all the drug transporters based on KEGG Orthology groups were found in the infant/elderly-enriched cluster, perhaps due to the frequent antibiotic treatment of infants and the elderly compared with adults.

In addition, elderly people are known to have decreased intestinal function relative to younger people, which affects digestion, nutrient absorption and immune activity [45, 46] and may also impact the microbiota composition [47, 48]. We found that certain oral bacteria, such as *Porphyromonas, Treponema, Fusobacterium* and *Pseudoramibacter*, which have difficulty reaching the intestinal tract due to barriers such as gastric juice and bile acid, were enriched in the elderly-associated CAGs (Additional file 6). Therefore, the decline in gastrointestinal tract functionality in the elderly may also lead to significant changes in gut microbiota.

## Conclusion

In conclusion, we provide a description of the changes in gut microbiota with age, thus illustrating the long trajectory throughout human life. Our results indicate some patterns and transition points in gut microbiota composition with age. The gut microbiota in subjects younger than 20 years changed with age as it matured, and that of subjects older than 70 years changed again into the elderly type. In addition, the transporter property prediction results suggested that nutrients in the gut might play an important role in changing the gut microbiota composition with age.

Our findings help clarify the gut microbiota composition in a healthy population at each age period. Further analyses investigating lifestyle traits or prospective cohorts focused on subjects who appear to have a gut microbiota typical of an age group older than their matched age would be valuable for revealing the relationships between gut microbiota and host health, including the aging process.

## Methods

### Subjects

Fecal samples were collected from a total of 367 community-dwelling Japanese volunteers (one sample per subject, except for two samples from one boy and one girl collected at preweaning and weaning and three samples from one girl at preweaning, weaning and 5 years of age) between 0 and 104 years of age (157 men and 210 women). Subjects over 80 years of age were directly recruited by the authors to confirm that they were community dwellers. The distribution of subjects according to age and individual data are shown in Table 1 and Additional file 12. No significant differences in gender distribution were observed among the age groups (Fisher’s test, *p* = 0.997). Fecal samples were collected from subjects that participated in three different studies. Two study protocols were for the collection of feces from subjects aged 21–65 years old or from community-dwelling elderly individuals and were approved by the Local Ethics Committee of the nonprofit organization Japan Health Promotion Supporting Network (Wakayama, Japan). The third study protocol was for the collection of feces from subjects aged 0–100 years old and was approved by the ethics committee of Kensyou-kai Incorporated Medical Institution (Osaka, Japan). Written informed consent was obtained from all subjects or from their legal guardians or relatives.

**Table 1:** Sample distribution

Group	Age		Number of samples	(Male/Female)
	Segmentation	(Mean ± SD)		
1	Preweaning	(0.3 ± 0.1)	14	(7/7)
2	Weaning	(0.8 ± 0.4)	12	(6/6)
3	Weaned-3 years old	(2.4 ± 0.6)	18	(10/8)
4	4–9 years old	(6.1 ± 1.9)	14	(6/8)
10	10–19 years old	(14.1 ± 3.6)	10	(7/3)
20	20–29 years old	(25.9 ± 2.7)	40	(16/24)
30	30–39 years old	(33.9 ± 2.3)	88	(45/43)
40	40–49 years old	(43.8 ± 3.1)	34	(13/21)
50	50–59 years old	(53.3 ± 2.6)	25	(12/13)
60	60–69 years old	(63 ± 2.7)	28	(11/17)
70	70–79 years old	(76.8 ± 2.1)	15	(5/10)
80	80–89 years old	(83.3 ± 2.4)	48	(16/32)
90	90–99 years old	(94.2 ± 2.7)	19	(4/15)
100	Over 100 years old	(101.3 ± 1.8)	6	(0/6)
	Sum		371	(158/213)

The mean (± SD) age of the entire cohort was 44.3 ± 28.6 years

Gut microbiota were analyzed for one sample per subject, except for two samples from one boy and one girl at preweaning and weaning and three samples from one girl at preweaning, weaning and 5 years of age

To identify the sequential changes in gut microbiota composition that occur with age, subjects were divided into 10-year age groups, except for subjects aged less than 10 years, who were divided into four groups: preweaning, weaning, weaned to 3 years old and 4–9 years old.

### Sampling

Fresh fecal samples were transferred by the subjects into tubes and immediately enclosed in plastic bags containing AnaeroPouch (Mitsubishi Gas Chemical, Tokyo, Japan) to create an anaerobic environment.

### Storage

The fecal samples collected from subjects younger than 80 years were stored at −20 °C for three days at most and were transported to the laboratory by logistics companies. The samples collected from subjects older than 80 years were stored at −20 °C and then delivered to the laboratory by the study authors within one day. Immediately upon receipt, the fecal samples were stored at −80 °C until the day of analysis.

### DNA extraction

A total of 20 mg of each fecal sample was collected from three regions (upper, middle and lower) and mixed well, and DNA was extracted using the bead-beating method as previously described [49]. After centrifugation at 14,000 × g for 5 min, 400 μl of the supernatant was extracted with phenol-chloroform, and 250 μl of the supernatant was precipitated with isopropanol. Purified DNA was suspended in 2,000 μl of Tris-EDTA buffer (pH 8•0).

### Sequencing and data processing

The V3-V4 region of the bacterial 16S rRNA gene was then amplified by PCR with the TaKaRa Ex Taq HS Kit (TaKaRa Bio, Shiga, Japan) and the primer sets Tru357F (5′-CGCTCTTCCGATCTCTGTACGGRAGGCAGCAG-3′) and Tru806R (5′-CGCTCTTCCGATCTGACGGACTACHVGGGTWTCTAAT-3′). Each 1-μl sample of DNA, at a concentration of approximately 10–200 ng/μl as measured using a Nanodrop 2000 (Thermo Fisher Scientific, Waltham, MA, USA), was amplified in triplicate using the following protocol: preheating at 94 °C for 3 min; 20 cycles of denaturation at 94 °C for 30 s, annealing at 50 °C for 30 s and extension at 72 °C for 30 s; and a final terminal extension at 72 °C for 10 min. After verifying the amplified DNA based on PCR product size using the QIAxcel system (Qiagen, Valencia, CA, USA), the triplicate samples were combined. A 1-μl sample of the combined PCR products was amplified using the following barcoded primers adapted for Illumina MiSeq: Fwd 5′-AATGATACGGCGACCACCGAGATCTACACXXXXXXXXACACTCTTTCCCTACACGACGCTCTTCCGATCTCTG-3′ and Rev 5′-CAAGCAGAAGACGGCATACGAGATXXXXXXXXGTGACTGGAGTTCAGACGTGTGCTCTTCCGATCTGAC-3′, where X represents a barcode base. The DNA was amplified according to the protocol described above except that only 8 cycles were performed. After validating the 2^nd^ amplified DNA product using the QIAxcel system, the PCR products were purified using a QIAquick 96 PCR Purification Kit (Qiagen, Valencia, CA, USA) according to the manufacturer’s protocol. The purified products were quantified using a Quant-iT PicoGreen dsDNA Assay Kit (Life Technologies, Carlsbad, CA, USA). Subsequently, equal amounts of the amplicons from multiple samples were pooled, and primer dimers were removed using a GeneRead Size Selection Kit (Qiagen, Valencia, CA, USA). The pooled libraries were sequenced using an Illumina MiSeq instrument with a MiSeq v3 Reagent Kit (Illumina, Inc., San Diego, CA, USA).

After removing sequences consistent with data from the Genome Reference Consortium human build 37 (GRCh37) or PhiX 174 from the raw Illumina paired-end reads, the 3′ region of each read with a PHRED quality score of less than 17 was trimmed. Trimmed reads less than 150 bp in length with an average quality score of less than 25 or those lacking paired reads were also removed. The reads that passed the quality filters were combined using the fastq-join script in EA-Utils (version 1.1.2–537) [50].

### Taxonomic analysis

The sequences were analyzed using the QIIME software package version 1.8.0 [51, 52] (http://qiime.org/).

Potential chimeric sequences were removed using UCHIME, assigned to operational taxonomic units (OTUs) using Open-reference OTU picking [53] with a 97 % threshold of pairwise identity, and then classified taxonomically using the Greengenes reference database (http://greengenes.secondgenome.com/downloads/database/13_5) [54]. An advantage of using the open reference method is that it minimizes spurious hits of sequencing reads to taxa that are not present in the gut; as a result, the number of obtained OTUs was lower than that in previous reports.

### Diversity analysis

The microbial diversity within each age-segmented group [alpha diversity including Chao1, number of observed species (the number of OTUs), phylogenetic distance (PD) whole tree, and Shannon diversity index] and the distances between subjects (UniFrac distance as beta diversity) were estimated using QIIME version 1.8.0 software.

### Clustering analysis

Hierarchical analysis was performed using the hclust function in R package 3.2.1. Distances based on the squared Euclidean distance were calculated for input into an agglomerative algorithm through Ward’s method. The population densities (z-scores) of genera scaled by color are displayed together with a dendrogram of bacterial genera in a heat map.

### Bacterial co-abundance groups (CAGs)

All genus-like-level groups, except for an unidentified group, were entered into this analysis. CAGs were defined by a heat plot showing Kendall correlations between genera clustered by Pearson’s correlation coefficient and Ward’s linkage hierarchical clustering in R using the Made4 package [55] as previously described [42]. The transition type of each CAG with aging indicates the sum of z-scores converted from the relative abundance of genera belonging to the same CAG. Network plots highlighting correlative relationships were visualized using Cytoscape version 3.2.1 [56]. These associations were controlled for multiple testing using the *q*-value method, and only those with a false discovery rate <0.05 were retained. The cut-off for line in the Wiggum plots was set at an absolute coefficient value of greater than 0.3.

### Bacterial strains and culture conditions

Cultivable strains were selected based on the Wiggum plot results and were obtained from public culture collections (Additional file 16). All the strains were pre-cultured at 37 °C for 16 h under anaerobic conditions in Gifu Anaerobic Medium (GAM) broth (Nissui Seiyaku Co. Ltd., Tokyo, Japan). Then, approximately 1 × 10^8^ cell in the pre-cultures, which were calculated by a density of McFarland, were added to GAM broth and incubated at 37 °C for 5 h under anaerobic conditions. After this culture period, the microorganisms were collected by centrifugation at 10,000 × g and DNA was extracted by the same method as described in the DNA extraction section. Experiments were performed in triplicate.

### Real-time PCR for quantitative determination of cell number

Real-time PCR was performed using an ABI PRISM 7500 Fast Real-time PCR System (Life Technologies, Carlsbad, CA, USA) with SYBR Premix Ex Taq (TaKaRa Bio Inc, Shiga, Japan). The primer sets are shown in Additional file 17 [57–60]. The amplification program consisted of one cycle of 94 °C for 10 s, followed by 40 cycles of 94 °C for 5 s, the appropriate annealing temperature for 30 s and 72 °C for 30 s. Fluorescent products were detected during the last step of each cycle. Melting curves were obtained by heating from 60 to 95 °C in 0.2 °C/s increments with continuous fluorescence collection.

### Phylogenetic investigation of communities by reconstruction of unobserved states (PICRUSt) analysis

PICRUSt analysis was performed to predict the relative abundance of transporter genes [61]. Independent of the taxonomic analysis, 97 % of the OTUs were picked using a closed-reference OTU picking protocol (QIIME 1.8.0 [51, 52]) and the Greengenes database (database/13_8) [54] pre-clustered at 97 % identity. The obtained OTU table was normalized by 16S rRNA copy number, and functional genes were predicted from the Kyoto Encyclopedia of Genes and Genomes (KEGG) catalogue [62].

### Statistical analysis

The gender distribution of subjects and intergroup differences at the genus level in each subcluster were analyzed by Fisher’s test and the Mann-Whitney *U* test, respectively, using SPSS version 23.0 statistical software (IBM, Armonk, NY, USA). Intergroup differences at the phylum, class, order, family and genus level in each cluster were analyzed by the linear discriminant analysis (LDA) effect size (LEfSe) method [63] with default settings on website (https://huttenhower.sph.harvard.edu/galaxy/root). Permutational MANOVA [64] was performed to test for significant differences in CAGs using the vegan package in R. Values of *p* < 0.05 were considered statistically significant.

### Data deposition

DNA sequences corresponding to the 16S rRNA gene data have been deposited in DDBJ under accession number DRA004160.

## Additional files

Additional file 1: UniFrac clustering for gender distribution. (A) Unweighted and (B) weighted UniFrac PCoA of gut microbiota from 371 samples collected from the infant to the centenarian stage. Male and female subjects are displayed as blue and red, respectively. (PDF 155 kb) Additional file 2: Relative abundance of four predominant phyla. The box-plot indicates the interquartile range (IQR) of the relative abundance of each phylum at each age stage. (PDF 211 kb) Additional file 3: Relative abundance of each microbiota at genus level. (XLSX 873 kb) Additional file 4: Definition of bacterial co-abundance groups (CAGs). CAGs were defined by a heat plot showing Kendall correlations between genera clustered by Pearson’s correlation coefficient and Ward’s linkage hierarchical clustering. The colors within the clustering indicate the nine CAGs as shown in Fig. 3. (PDF 148 kb) Additional file 5: Network plot highlighting relationships between genera in nine CAGs. The colors of each node indicate the nine CAGs as shown in Fig. 3. Circle size indicates genus abundance. Pink and blue lines show significant positive and negative correlations between two bacterial genera with an absolute coefficient value greater than 0.3. (PDF 401 kb) Additional file 6: Bacterial groups at genus-like level in each CAG. (XLSX 863 kb) Additional file 7: Cell number of bacteria in vitro assay. (XLSX 855 kb) Additional file 8: Hierarchical Ward’s linkage clustering based on bacterial proportion at the genus level. Age-related groups (Infant, Elderly1, Elderly2, Adult1 and Adult2) were revealed through Ward’s linkage clustering using the squared Euclidean distance. The population densities (z-score) of genera scaled by color are displayed together with a dendrogram of bacterial genera in a heat map. The colors within the horizontal and vertical clustering represent age-segmented groups as shown in Fig. 1 and phylum as shown in Fig. 2. (PDF 245 kb) Additional file 9: LEfSe results on human microbiota in infant, adult and elderly cluster. (A) Histogram of the LDA scores computed for features differentially abundant between three age-related clusters. (B) Taxonomic representation of statistically differences between three age-related clusters. Differences are represented in the color of the most abundant class (yellow non-significant). Each circle’s diameter is proportional to the taxon’s abundance. (PDF 400 kb) Additional file 10: Taxa that are found in more than 50 % of the subjects in any cluster (shown in Additional file 8) with significantly difference between adult 1 and adult 2 clusters. (XLSX 857 kb) Additional file 11: Taxa that are found in more than 50 % of the subjects in any cluster (shown in Additional file 8) with significantly difference between elderly 1 and elderly 2 clusters. (XLSX 859 kb) Additional file 12: Individual data. (XLSX 875 kb) Additional file 13: Hierarchical Ward’s linkage clustering based on the proportion of transporter genes predicted by PICRUSt. Age-related groups (adult-enriched and infant/elderly-enriched clusters) were revealed by Ward’s linkage clustering using the squared Euclidean distance. The population densities (z-score) of the transporters scaled by color are displayed together with a dendrogram of the transporters in a heat map. The colors within the horizontal clustering represent the age-segmented groups as shown in Fig. 1 The color code for the vertical clustering indicates KEGG Orthology (KO) as follows: white, ABC Transporters, Eukaryotic Type; yellow, ABC Transporters, Prokaryotic Type; blue, Solute Carrier Family (SLC); orange, Major Facilitator Superfamily (MFS); red, Phosphotransferase System (PTS); and green, Other Transporters. (PDF 209 kb) Additional file 14: Relative abundance of predicted D-Xylose transporter (KEGG module: M00215). The KEGG module M00215 consists of three KO entries, K10543, K10544 and K10545. Each number indicates a group as shown in Table 1. Box-plots show the interquartile range (IQR) of the relative abundance of the predicted D-Xylose transporter. Open circles indicate outliers from 1.5- to 3.0-fold IQR. (PDF 96 kb) Additional file 15: List of predicted transporters in Additional file 13. (XLSX 879 kb) Additional file 16: Bacterial strains used in this study. (XLSX 855 kb) Additional file 17: PCR primers for detection of each species. (XLSX 857 kb)

## Abbreviations

CAG: co-abundance group
DNA: deoxyribonucleic acid
OTU: operational taxonomic units
PCR: polymerase chain reaction
QIIME: quantitative insights into microbial ecology
rRNA: ribosomal ribonucleic acid
