# Supplementary figures and images for: Age-related changes in gut microbiota composition from newborn to centenarian: a cross-sectional study
Source: BMC Microbiol. 2016 May 25;16:90. doi: 10.1186/s12866-016-0708-5 (PMC4879732; doi:10.1186/s12866-016-0708-5)

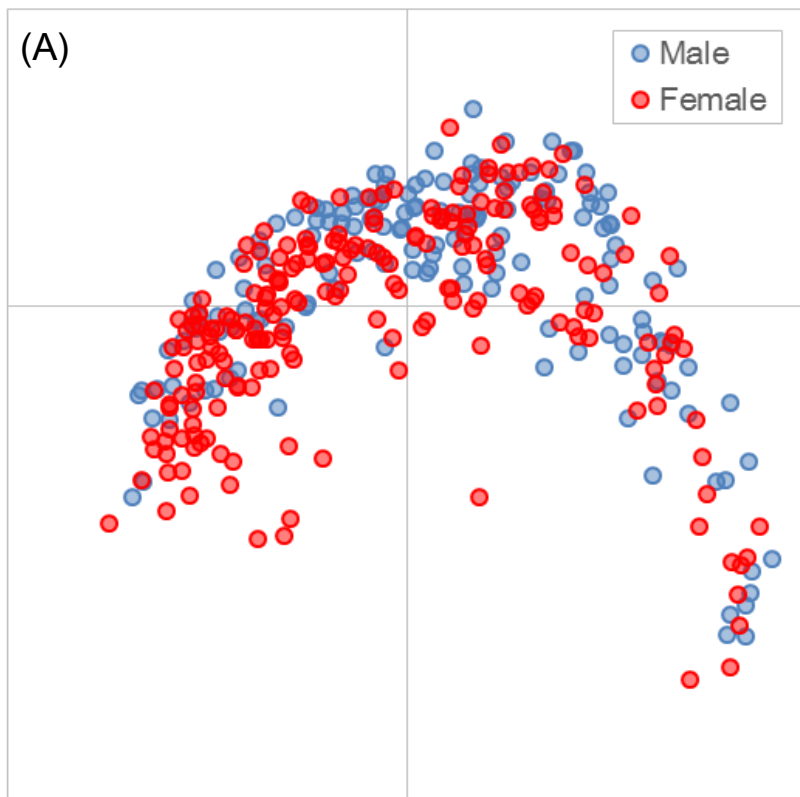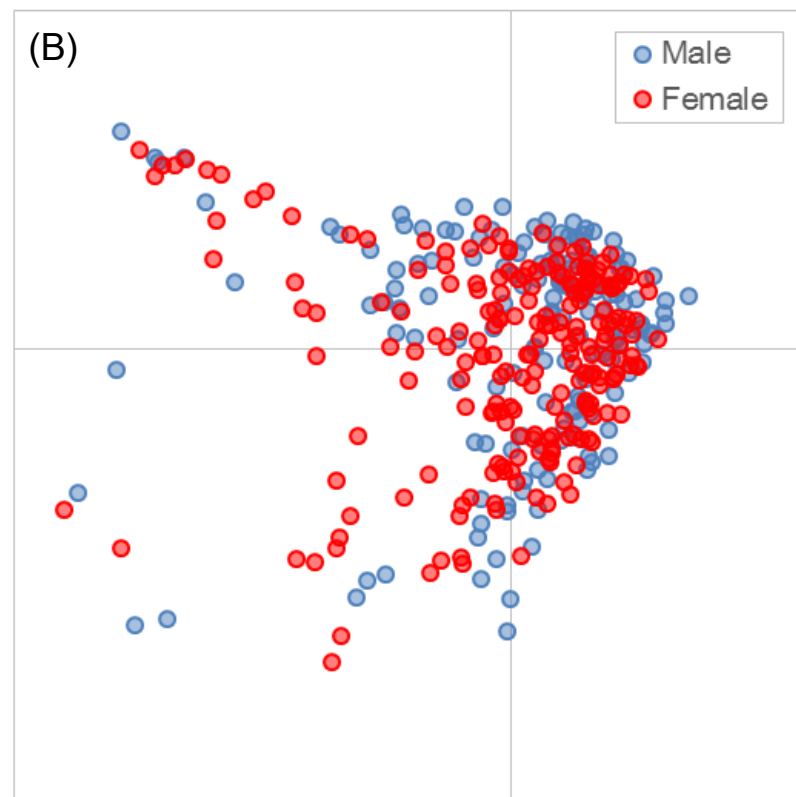

Supplement: Additional file 1: — UniFrac clustering for gender distribution. (A) Unweighted and (B) weighted UniFrac PCoA of gut microbiota from 371 samples collected from the infant to the centenarian stage. Male and female subjects are displayed as blue and red, respectively. (PDF 155 kb) [file 12866_2016_708_MOESM1_ESM.pdf]

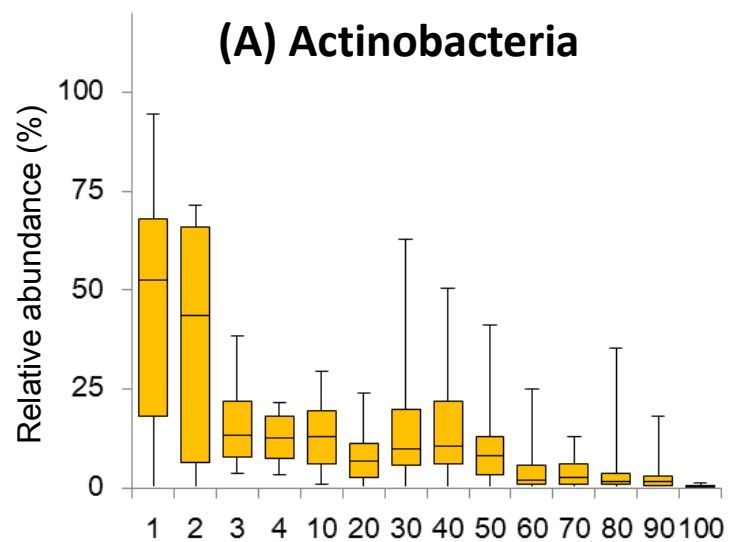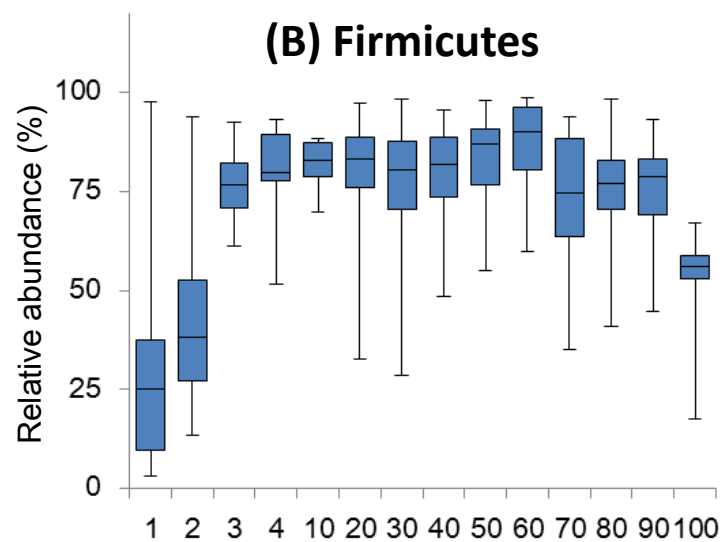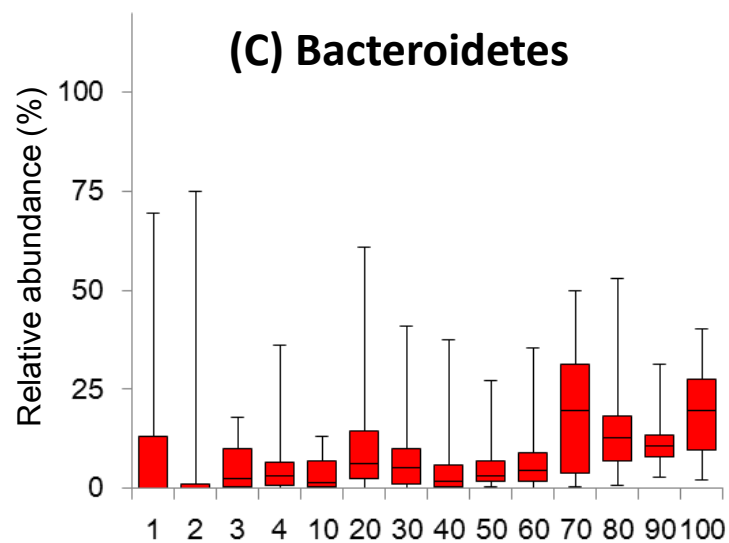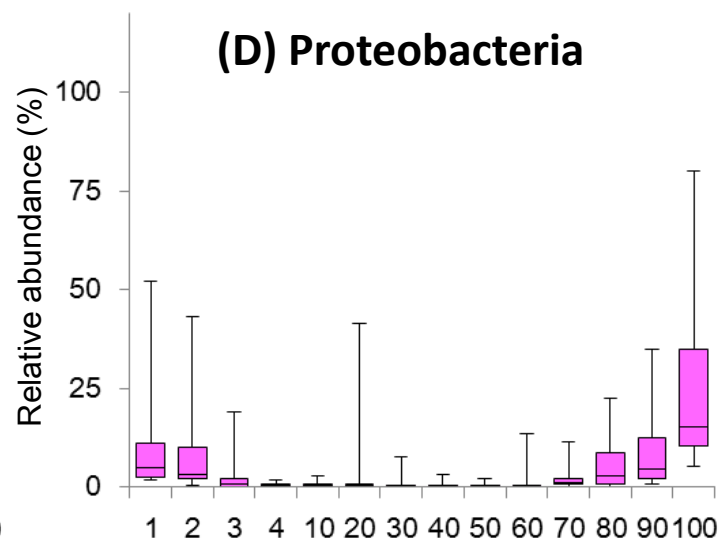

Supplement: Additional file 2: — Relative abundance of four predominant phyla. The box-plot indicates the interquartile range (IQR) of the relative abundance of each phylum at each age stage. (PDF 211 kb) [file 12866_2016_708_MOESM2_ESM.pdf]

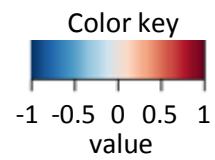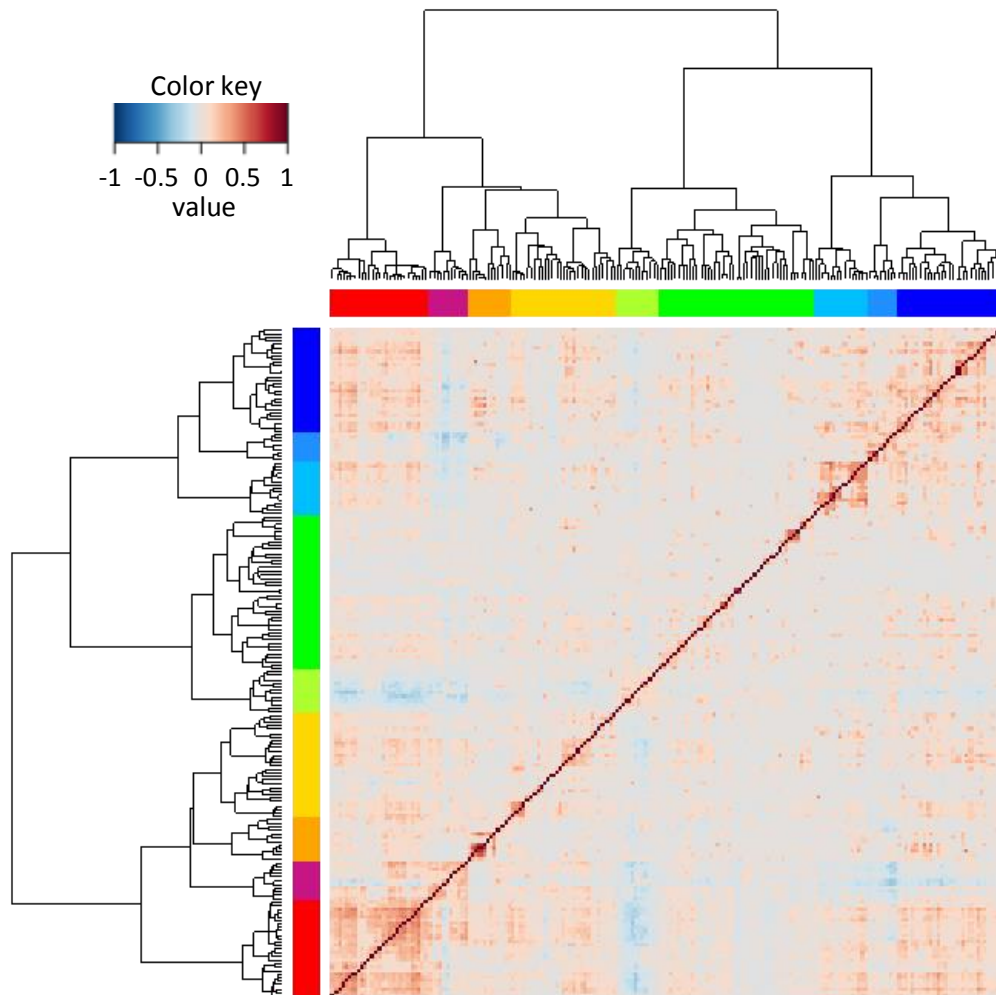

Supplement: Additional file 4: — Definition of bacterial co-abundance groups (CAGs). CAGs were defined by a heat plot showing Kendall correlations between genera clustered by Pearson’s correlation coefficient and Ward’s linkage hierarchical clustering. The colors within the clustering indicate the nine CAGs as shown in Fig. 3. (PDF 148 kb) [file 12866_2016_708_MOESM4_ESM.pdf]

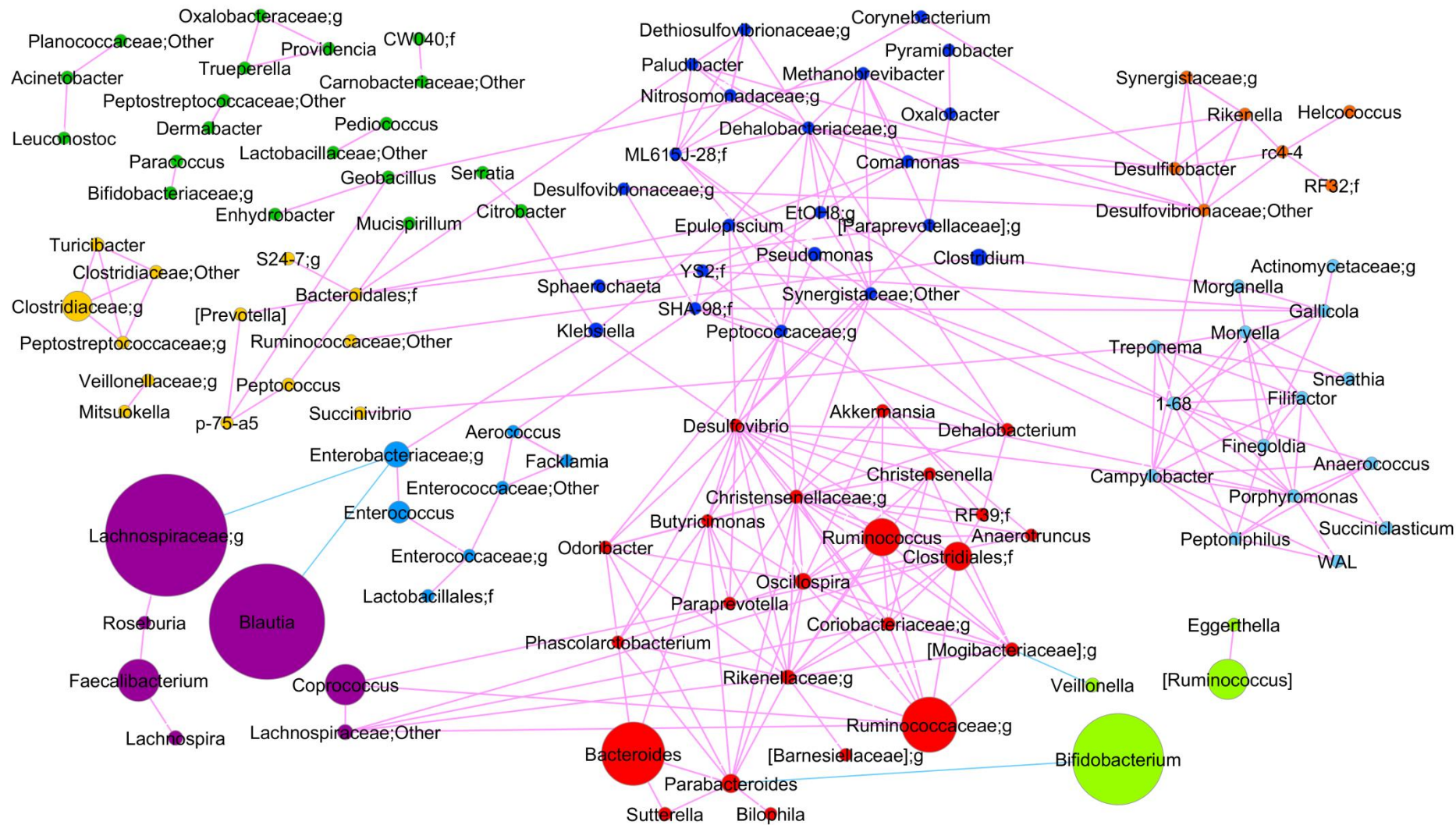

Supplement: Additional file 5: — Network plot highlighting relationships between genera in nine CAGs. The colors of each node indicate the nine CAGs as shown in Fig. 3. Circle size indicates genus abundance. Pink and blue lines show significant positive and negative correlations between two bacterial genera with an absolute coefficient value greater than 0.3. (PDF 401 kb) [file 12866_2016_708_MOESM5_ESM.pdf]

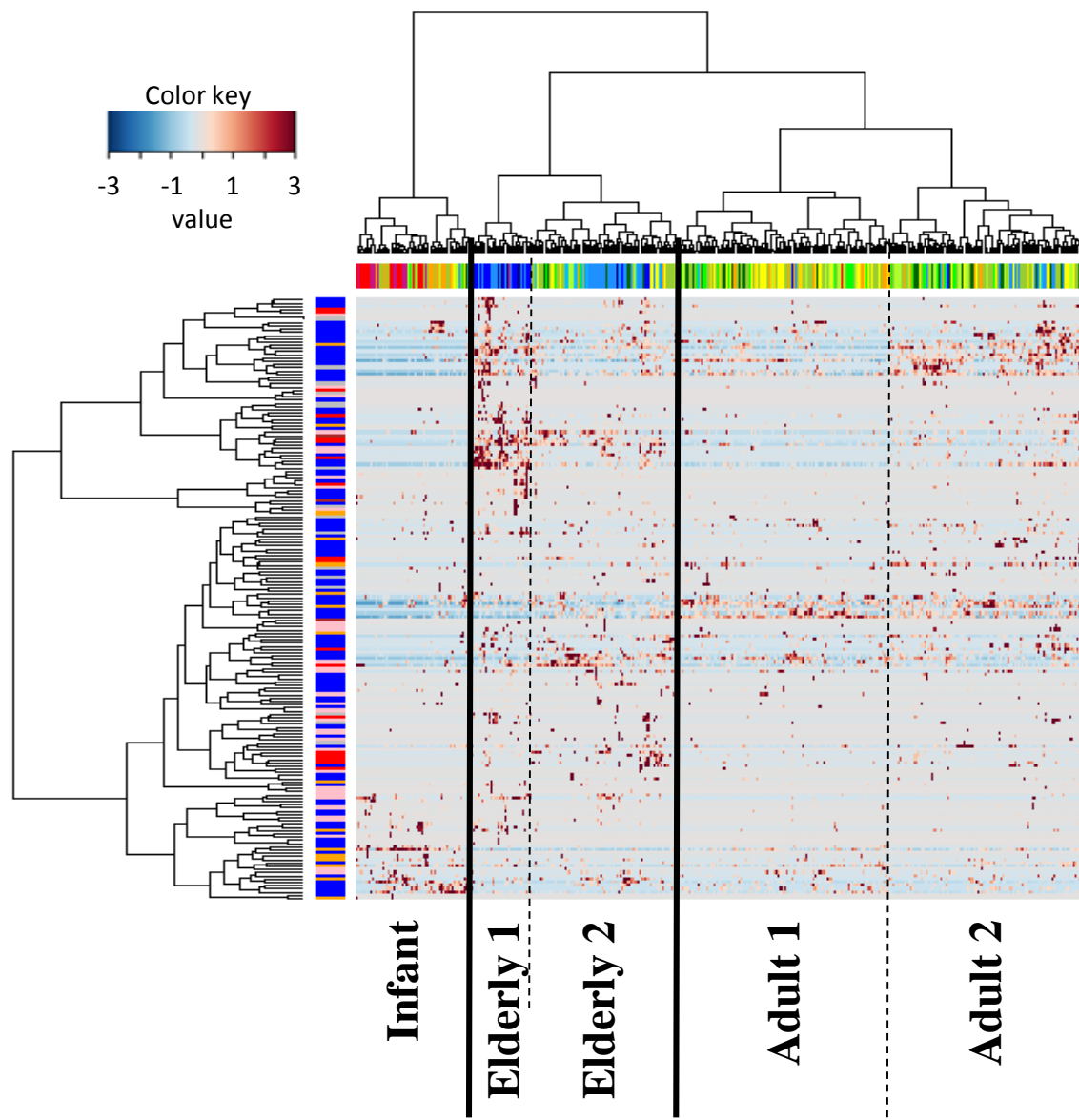

Supplement: Additional file 8: — Hierarchical Ward’s linkage clustering based on bacterial proportion at the genus level. Age-related groups (Infant, Elderly1, Elderly2, Adult1 and Adult2) were revealed through Ward’s linkage clustering using the squared Euclidean distance. The population densities (z-score) of genera scaled by color are displayed together with a dendrogram of bacterial genera in a heat map. The colors within the horizontal and vertical clustering represent age-segmented groups as shown in Fig. 1 and phylum as shown in Fig. 2. (PDF 245 kb) [file 12866_2016_708_MOESM8_ESM.pdf]

(A)

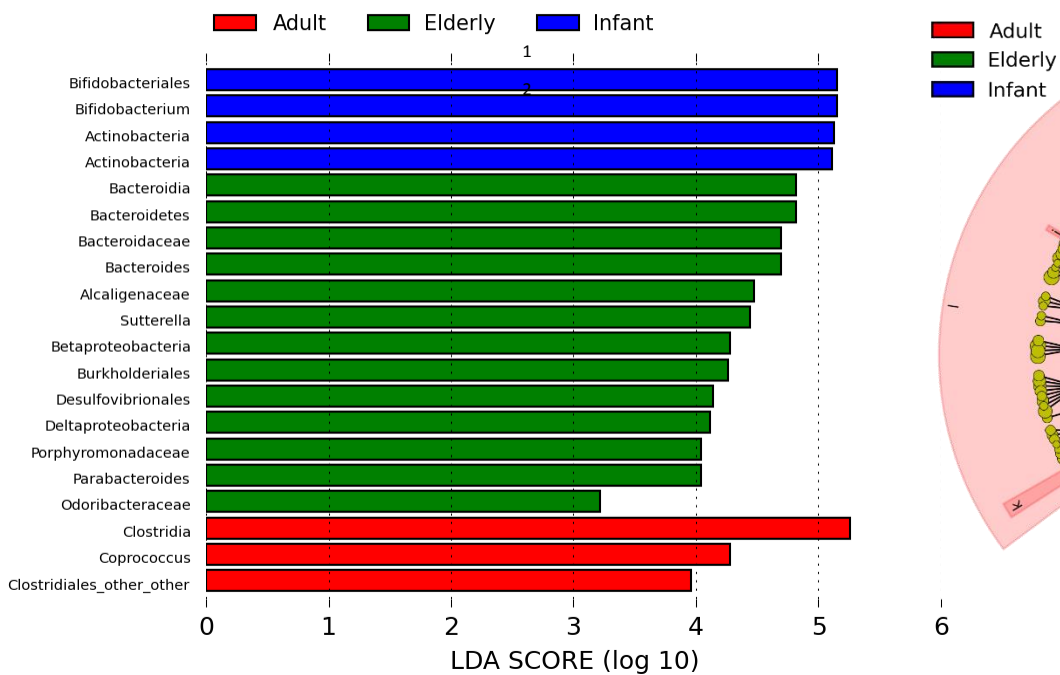

(B)

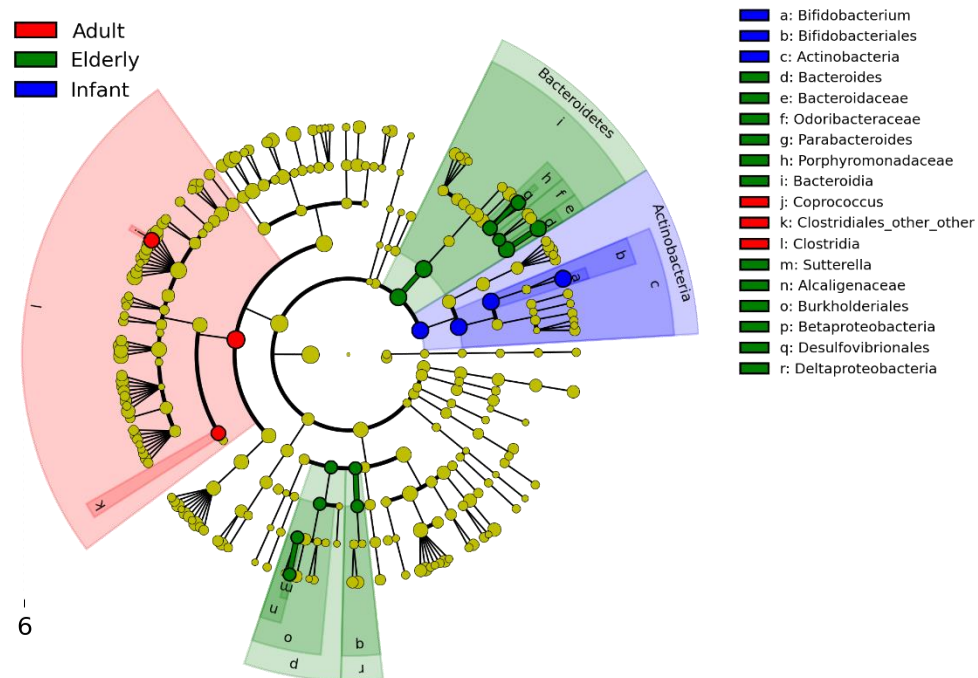

Supplement: Additional file 9: — LEfSe results on human microbiota in infant, adult and elderly cluster. (A) Histogram of the LDA scores computed for features differentially abundant between three age-related clusters. (B) Taxonomic representation of statistically differences between three age-related clusters. Differences are represented in the color of the most abundant class (yellow non-significant). Each circle’s diameter is proportional to the taxon’s abundance. (PDF 400 kb) [file 12866_2016_708_MOESM9_ESM.pdf]

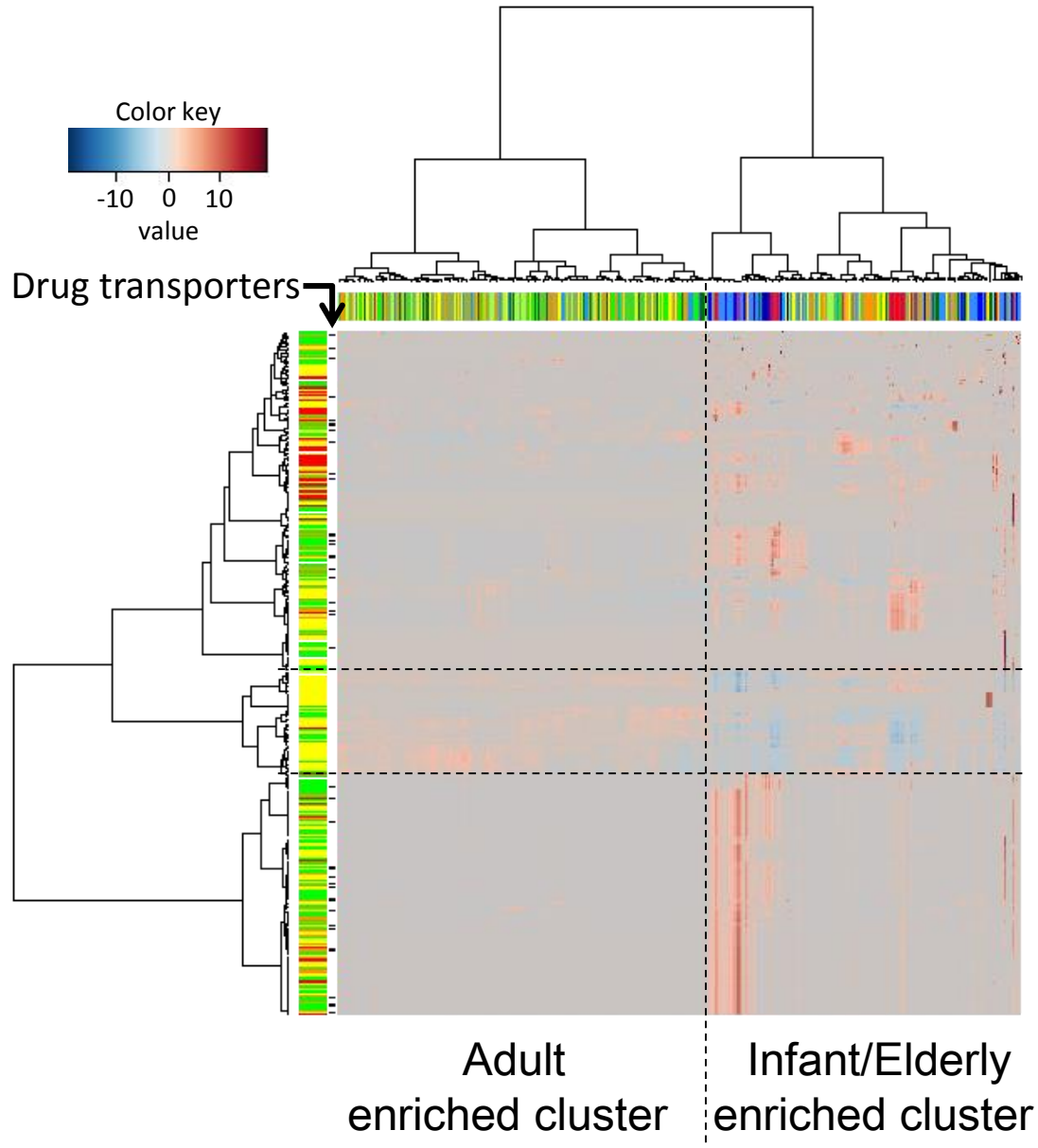

Supplement: Additional file 13: — Hierarchical Ward’s linkage clustering based on the proportion of transporter genes predicted by PICRUSt. Age-related groups (adult-enriched and infant/elderly-enriched clusters) were revealed by Ward’s linkage clustering using the squared Euclidean distance. The population densities (z-score) of the transporters scaled by color are displayed together with a dendrogram of the transporters in a heat map. The colors within the horizontal clustering represent the age-segmented groups as shown in Fig. 1 The color code for the vertical clustering indicates KEGG Orthology (KO) as follows: white, ABC Transporters, Eukaryotic Type; yellow, ABC Transporters, Prokaryotic Type; blue, Solute Carrier Family (SLC); orange, Major Facilitator Superfamily (MFS); red, Phosphotransferase System (PTS); and green, Other Transporters. (PDF 209 kb) [file 12866_2016_708_MOESM13_ESM.pdf]

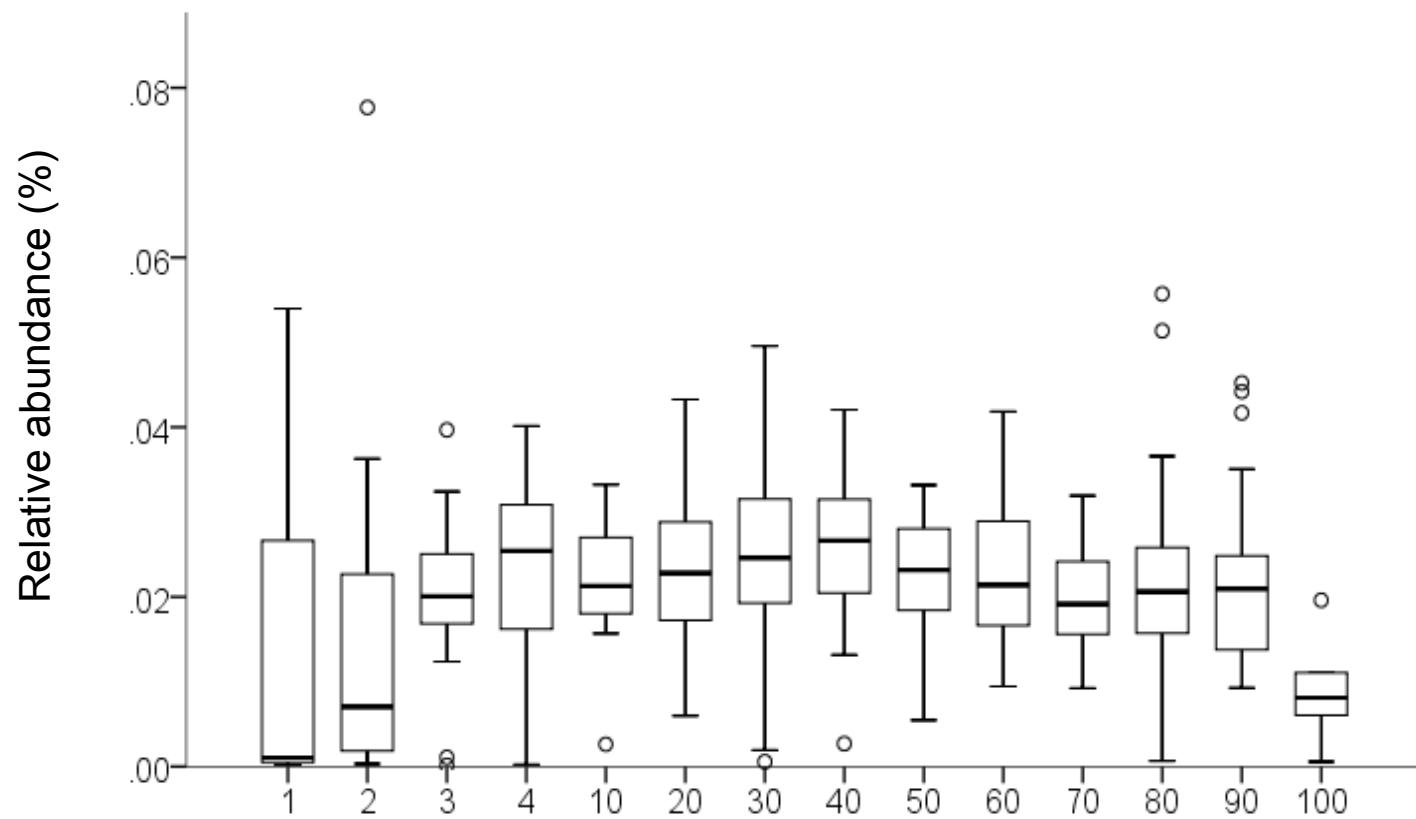

Supplement: Additional file 14: — Relative abundance of predicted D-Xylose transporter (KEGG module: M00215). The KEGG module M00215 consists of three KO entries, K10543, K10544 and K10545. Each number indicates a group as shown in Table 1. Box-plots show the interquartile range (IQR) of the relative abundance of the predicted D-Xylose transporter. Open circles indicate outliers from 1.5- to 3.0-fold IQR. (PDF 96 kb) [file 12866_2016_708_MOESM14_ESM.pdf]
